# Integrative Genomic Analysis Identifies MAGT1 as a Key Regulator of Proliferation and Poor Prognosis in Breast Cancer

**DOI:** 10.1155/humu/8673018

**Published:** 2026-04-17

**Authors:** Liwen Zhao, Zhe Song

**Affiliations:** ^1^ Department of Anesthesiology, The Second Affiliated Hospital, Hengyang Medical School, University of South China, Hengyang, Hunan, China, usc.edu.cn; ^2^ Department of Urology, The Second Affiliated Hospital, Hengyang Medical School, University of South China, Hengyang, Hunan, China, usc.edu.cn

**Keywords:** breast cancer, MAGT1, prognosis

## Abstract

**Objective:**

Magnesium transporter 1 (MAGT1) plays a crucial role in magnesium homeostasis and immune regulation, yet its clinical significance and functional role in breast cancer remain largely unexplored.

**Methods:**

The expression pattern and prognostic value of MAGT1 in breast cancer were analyzed using data from The Cancer Genome Atlas (TCGA) and validated by immunohistochemistry on a tissue microarray comprising 60 patient samples. Genomic alteration analysis of MAGT1 with BRCA clinical implications was performed. The biological functions of MAGT1 were investigated in vitro using MCF‐7 and MDA‐MB‐231 cell lines. MAGT1 expression was knocked down by siRNA, and its effects on cell proliferation, colony formation, DNA synthesis, migration, and invasion abilities were inhibited through MTT assays, colony formation assays, EdU assays, wound healing assays, and Transwell assays. Immune cell infiltration associated with MAGT1 expression was analyzed using bioinformatics tools.

**Results:**

MAGT1 was significantly overexpressed in breast cancer tissues compared with adjacent normal tissues. High MAGT1 expression was strongly associated with advanced tumor stage, poorer histological grade, and unfavorable patient prognosis, serving as an independent risk factor for overall survival. MAGT1 mutations were not statistically significantly associated with overall survival (OS) in breast cancer, but MAGT1 mutations were closely associated with ERBB2 and CDH1. Bioinformatic analysis revealed a correlation between MAGT1 expression and altered immune cell infiltration within the tumor microenvironment. In vitro functional assays demonstrated that silencing MAGT1 markedly inhibited the proliferative capacity, clonogenicity, migration, and invasion of breast cancer cells.

**Conclusion:**

Our findings indicate that MAGT1 is frequently upregulated in breast cancer and correlates with aggressive tumor behavior and poor clinical outcomes. MAGT1 promotes key oncogenic phenotypes in breast cancer cells and may influence the immune landscape, highlighting its potential as both a prognostic biomarker and a promising therapeutic target.

## 1. Introduction

According to the “Global Cancer Statistics 2020” report, breast cancer (BRCA) has surpassed lung cancer to become the most common malignant tumor among women, with approximately 2.3 million new cases. BRCA has become the fifth leading cause of cancer‐related deaths, with 685,000 deaths ranking it among the Top 5 in the world [1]. The three main factors affecting the clinical prognosis of BRCA patients are drug resistance, recurrence, and metastasis [2]. Even with adjuvant chemotherapy, the 5‐year survival rate for metastatic BRCA patients is less than 30% [3]. In recent years, although the diagnostic and treatment capabilities of BRCA have greatly improved, the number of deaths and overall survival (OS) rates of BRCA worldwide remain grim.

MAGT1 is a highly conserved, evolutionarily specific magnesium transporter expressed in all animals. It has been implicated in multienzyme complexes responsible for coupling N‐glycosylation to peptide substrates [4]. As a plasma membrane transporter highly selective for Mg^2+^, MAGT1 critically regulates intracellular free Mg^2+^ levels and plays an essential role in the timely activation of natural killer (NK) and CD8^+^ T cells [5, 6]. Consequently, loss‐of‐function mutations in MAGT1 lead to the rare primary immunodeficiency disorder XMEN syndrome (X‐linked MAGT1 deficiency with Epstein–Barr virus infection and neoplasia) [7]. Given the emerging link between Mg^2+^ homeostasis and oncogenic processes, accumulating evidence indicates that MAGT1 dysregulation contributes to tumorigenesis in multiple cancer types, potentially through shared mechanisms involving altered cation signaling and immune surveillance [8]. Similarly, colorectal cancer (CRC) tissues exhibit elevated MAGT1 expression, which positively correlates with advanced tumor stage and increased risk of lymphatic or distant metastasis [9]. Functionally, overexpression of MAGT1 in CRC cells confers resistance to azathioprine and promotes proliferation. It has been reported previously that knockdown of MAGT1 inhibits Ca^2+^ signaling in BRCA cells, thereby suppressing cell viability [10]. Although it has been demonstrated that MAGT1 knockdown significantly suppresses the proliferation of MCF‐7 BRCA cells, its expression in BRCA tissues and its clinical prognostic significance remain unreported [11]. To address this, we investigated the role of MAGT1 in regulating the proliferation and invasion of multiple BRCA cell lines. Our analysis of clinical specimens further revealed that MAGT1 expression was significantly upregulated in BRCA tissues compared with adjacent normal tissues. These results provide additional evidence that MAGT1 may be a critical prognostic indicator for BRCA.

In this study, we conducted a comprehensive analysis based on MAGT1 expression and relevant clinical pathological features in TCGA and found that MAGT1 expression is associated with OS prognosis and is also an independent prognostic marker affecting patient OS. Further immunoinfiltration analysis revealed a close correlation between MAGT1 expression and immune infiltration. Immunohistochemical results indicated high expression of MAGT1 in BRCA tissues. Knocking down MAGT1 significantly inhibited the proliferation and migration abilities of BRCA cells. The experimental methodology employed in this study was based on previously described protocols [12, 13]. Our results confirm that MAGT1 is a reliable biomarker for the prognosis of BRCA patients.

## 2. Materials and Methods

### 2.1. Data Source

We collected mRNA and clinical data from 1222 cases (1109 cases and 113 normal tissues) of BRCA from TCGA. Based on the median expression value of MAGT1, we divided the patients into two groups (high/low MAGT1 expression groups) and analyzed the relationship between MAGT1 and clinical pathological features as well as prognosis.

### 2.2. Differential Gene Expression Analysis

We used median scores to divide BRCA patients into low and high MAGT1 expression groups. Differential expression genes (DEGs) were analyzed using R software, with |(logFC)| > 1 and *p* < 0.05 as the thresholds [14]. Volcano plots and heat map visualization analyses were conducted for the DEGs

### 2.3. Gene Set Enrichment Analysis (GSEA)

GSEA and Kyoto Encyclopedia of Genes and Genomes (KEGG) pathways were used to explore the biological functions of MAGT1 in BRCA. Enrichment results were considered statistically significant when the false discovery rate (FDR) was < 0.25 and the adjusted *p* value was < 0.0

### 2.4. Genomic Alteration Analysis

The genomic characteristics and mutational signatures of MAGT1 across different tumor types were analyzed using the cBioPortal platform (https://www.cbioportal.org). Putative copy number alterations (CNAs) were identified using GISTIC to assess their relationship with MAGT1 mRNA expression levels. Mutation sites within MAGT1 were mapped onto a schematic diagram of its protein structure. The association between MAGT1 genetic alterations and prognosis in BRCA was assessed using the “Comparison/Survival” module. Additionally, we integrated pan‐cancer CNA data from the “mutation” module of the Gene Set Cancer Analysis (GSCA) database (http://bioinfo.life.hust.edu.cn/GSCA). BRCA patients were stratified into two groups based on the median expression level of MAGT1. The somatic mutation landscape of these groups was visualized using the R package “waterfalls” [15].

### 2.5. Immunoinfiltration Analysis

We utilized ssSEA to analyze the immune infiltration status in BRCA samples, and the GSVA package in R (http://www.biocondutor.org/package/release/bioc/html/GSVA.html) was employed to analyze the infiltration status of 22 types of immune cells [15, 16].

### 2.6. Cell Lines and Reagents

Human BRCA cell lines MCF7 (RRID : CVCL_0031), MDA‐MB‐231 (RRID : CVCL_0062), HCC1937 (RRID : CVCL_0290), and Hs‐578 T (RRID : CVCL_0332) and human normal breast epithelial cells MCF‐10A (RRID : CVCL_0598) were provided by the Cell Bank of the Chinese Academy of Sciences (Shanghai, China). All cell lines were authenticated by short tandem repeat (STR) profiling and confirmed to be free of mycoplasma contamination. All cell lines were cultured in 1640 or DMEM medium supplemented with 10% fetal bovine serum (FBS) and maintained in a humidified atmosphere with 5% carbon dioxide. One thousand, six hundred and forty or DMEM medium was purchased from Gibco, United States, and FBS was purchased from Prolab, China. Both cell lines underwent STR profiling prior to use. Verification through the ATCC database confirmed that neither MCF7, MDA‐MB‐231, HCC1937, Hs‐578 T, and MCF‐10A had been reported as misidentified or contaminated. The revised version will include a corresponding statement. Prior to commencing the experiment, mycoplasma contamination testing was performed on the MCF7 and MDA‐MB‐231 cell lines using the polymerase chain reaction (PCR) method, with both yielding negative results.

### 2.7. RNA Extraction and Real‐time PCR

Cells were treated with TRIzol (ThermoFisher, Massachusetts, United States) to extract total RNA. Reverse transcription was performed using the PrimeScript RT Master Mix (TaKaRa, Kyoto, Japan), followed by real‐time PCR. The specific primers for MAGT1 and GAPDH were as follows: MAGT1 forward 5 ^′^‐CGTCATGTTCACTGCTCTCCAAC‐3 ^′^ and reverse 5 ^′^‐CCTGTTGGTGAATGCACTGGAG‐3 ^′^, GAPDH forward 5 ^′^‐GTCTCCTCTGACTTCAACAGCG‐3 ^′^ and reverse 5 ^′^‐ACCACCCTGTTGCTGTAGCCAA‐3 ^′^.

### 2.8. Silencing MAGT1

BRCA cell lines MCF7 and MDA‐MB‐231 cells in good condition were seeded into 6‐well plates. Once the cells reached 30% confluence, take a clean sterile centrifuge tube, add 125 *μ*L of antibiotic‐free Opti‐MEM Medium to each well of the 6‐well plate to be transfected, add 2.5 *μ*g of MAGT1 lasmid DNA, and mix with a gentle pipette, and then add 4 *μ*L of Lipo8000 transfection reagent and mix with a gun by gently pipetting, taking special care not to vortex or centrifuge. The concentration of plasmid should be controlled at 2.5 *μ*g/*μ*L. After 48 h of infection, MCF‐7 and MDA‐MB‐231 cells were harvested for RT‐qPCR and western blot analysis the silencing efficiency.

### 2.9. MTT Assay

MCF7 and MDA‐MB‐231 cell mixtures were seeded into 96‐well plates with 3000 cells per well, and 200 *μ*L of sterile phosphate‐buffered saline (PBS) was added to the edge wells. The plates were then placed in a cell culture incubator and incubated for different time gradients as required by the experiment. After the specified incubation period, 20 *μ*L of MTT solution (5 mg/mL, Solarbio, Beijing, China) was added to each well, and the cells were further incubated for 4 h. The MTT solution in each well was gently aspirated, and then 150 *μ*L of DMSO was added to each well and shaken gently for 10 min to completely dissolve the formazan crystals. The plates were then placed in a microplate reader at 570 nm to read the OD values of each well.

### 2.10. Wound Healing Assay

MCF7 and MDA‐MB‐231 cells were seeded into 6‐well plates and cultured overnight. When the cell density reached 90%, a scratch was made on the cell surface using a 1000 *μ*l pipette tip. Photographs of the scratch were taken using an inverted microscope (CKX31, Olympus, Tokyo, Japan). Subsequently, the cells were treated with serum‐free medium, and after 48 h of treatment, photographs of the scratch were taken at the same position using the microscope.

### 2.11. Colony Formation Assays

The MCF7 and MDA‐MB‐231 cells in the logarithmic growth phase (80%–90%) were subjected to trypsin digestion, followed by centrifugation and resuspension to achieve a concentration of 1000 cells/mL. Subsequently, 200 cells were seeded into each well of a 6‐well plate and permitted to adhere for 48 h before being replaced with fresh complete medium. Culturing was terminated after 10–14 days upon the visible formation of colonies. The cells were then washed with PBS, fixed using 4% paraformaldehyde, stained with 0.1% crystal violet, and subjected to photographic documentation for subsequent colony analysis.

### 2.12. Transwell Assay

The matrix gel working solution was prepared by mixing precooled DMEM medium and matrix gel (1:5 ratio). Fifty microliters of the matrix gel working solution was aspirated into the bottom of each well of the Transwell. The Transwell and 24‐well plates were incubated in the incubator for 2 h, and then checked to confirm if the matrix gel had solidified. The cell concentration was adjusted to 2.5 × 10^6^ cells/mL using cell counting. Two hundred microliters of cell suspension was aspirated into each Transwell, and 500 *μ*L of 20% complete culture medium was added to the wells to ensure contact between the cells and the liquid in the wells. The cells were subjected to migration experiments for 24 h and invasion experiments for 36 h. After incubation, the Transwell plates were removed, washed with PBS three times for 5 min each, fixed with 4% paraformaldehyde for 20 min, washed again with PBS three times for 5 min each, air‐dried, stained with crystal violet for 30 min, washed with PBS three times for 5 min each, air‐dried, and then photographed.

### 2.13. EdU Assay

Logarithmic growth phase cells were digested and centrifuged, and each group of cells was adjusted to 3 × 10^4^ cells/mL using basal culture medium. One hundred microliters of the cell suspension was added to each well of five replicate wells in a plate, and cell adhesion was observed overnight. Each well was then treated with 100 *μ*L of EdU working solution and incubated in a cell culture incubator for 2 h. After washing with PBS once, the cells were fixed with 4% paraformaldehyde at room temperature for 30 min. PBS containing 0.3% TritonX was used to permeabilize the cells, and the plate was left at room temperature for 15 min. Click reaction solution was prepared according to the kit instructions and added to the plate, followed by incubation at room temperature in the dark for 30 min. A 1× Hoechst solution was prepared for nuclear staining and incubated at room temperature in the dark for 10 min. After washing with PBS three times, images were captured under a fluorescence microscope, and data were analyzed.

### 2.14. Western Blot

Whole‐cell or tissue lysates were prepared using precooled NP‐40 buffer mixed with protease and phosphatase inhibitors (Roche, Indiana, United States), followed by centrifugation at 14,000 × g for 10 min at 4°C. The protein lysates were separated by SDS‐PAGE and transferred onto a polyvinylidene fluoride (PVDF) membrane (Merck, Darmstadt, Germany). The membrane was incubated in 1× TBST buffer containing 5% nonfat milk powder at room temperature for 2 h. Primary antibodies were incubated with the PVDF membrane, followed by washing with 1× TBST and detection using HRP‐conjugated secondary antibodies. Signal detection was performed using the Western ECL Substrate Kit (Bio‐Rad, Hercules, California, United States). The following antibodies were used for immunoblotting: MAGT1 (67537‐1‐Ig 1:1000) and *β*‐actin (81115‐1‐RR 1:10000) from Proteintech (Wuhan, China). All western blot analyses were performed at least three times.

### 2.15. Immunohistochemistry

Sixty paraffin‐embedded BRCA tissue specimens were collected from the Pathology Department of the Second Affiliated Hospital of University of South China. The protein expression of MAGT1 in BRCA tissues was detected according to the standard immunohistochemistry process. MAGT1 antibody (67537‐1‐Ig, 1:100) was used for incubation according to the kit instructions. Immunohistochemical staining scores were evaluated by two pathologists with the title of associate chief physician or above. The percentage score of positively stained cells was assigned based on the following categories: 1 point for ≤ 25% positive tumor cells; 2 points for 26%–50% positive tumor cells; 3 points for 51%–75% positive tumor cells; and 4 points for > 75% positive tumor cells. The staining intensity score ranged from 0 to 3: 0 for no staining, 1 for weak staining, 2 for moderate staining, and 3 for strong staining. The weighted score for each patient was obtained by multiplying the percentage score by the staining intensity score. Based on the distribution of weighted scores, a score of 8–12 was considered a high expression level, whereas a score of 0–7 was considered a low expression level. The correlation between MAGT1 expression and clinicopathological features of BRCA patients was statistically analyzed.

### 2.16. Statistical Analysis

Wilcoxon rank‐sum and Wilcoxon signed‐rank tests were used to analyze the expression of MAGT1 in unpaired and paired samples, respectively. The RMSR package in R software was used to generate column charts of clinical variables related to MAGT1 in BRCA. The Kaplan–Meier analysis method was used to evaluate the prognostic value of MAGT1 in BRCA. Univariate and multivariate Cox regression analyses were conducted to determine the prognostic factors of BRCA. All statistical analyses were performed using R software (Version 4.0.3) to generate plots. A *p* value less than 0.05 was considered statistically significant.

## 3. Results

### 3.1. Association of MAGT1 With Clinical Pathological Features and Poor Prognosis in BRCA

We initially analyzed the expression of MAGT1 at the mRNA level across various cancers (Figure 1a), revealing a significant increase in MAGT1 expression in BRCA patients (Figure 1b,c). Kaplan–Meier survival analysis indicated that MAGT1 expression affected the OS, disease‐specific survival (DSS), and progression‐free interval (PFI) of BRCA patients (Figure 1d, 1e, 1f). The area under the ROC curve was 0.779 (Figure 1g). The qPCR and western blot experiments confirmed that MAGT1 expression in BRCA cells was significantly higher than in normal breast epithelial cells (Figure 1h,i). MCF‐7 and MDA‐MB‐231 cells were selected for subsequent functional experiments. Our subsequent analysis of GEO datasets revealed that MAGT1 expression is significantly correlated with OS (Figure S1). Specifically, analysis of the BRCA datasets GSE131769, GSE47994, and GSE42568 showed that high MAGT1 expression is associated with poor prognosis (Figure S1A–C).

Figure 1Association between the MAGT1 expression levels and clinical characteristics in patients with breast cancer. (a) MAGT1 expression in pan‐cancer. (b) MAGT1 expression in nonconfigurable tissue in breast cancer. (c) MAGT1 expression in configurable tissue in breast cancer. (d–f) The high levels of MAGT1 exhibited worse overall survival, DSS, and progress‐free interval in the patients with breast cancer. (g) The time ROC analysis was used MAGT1expression. (h–i) MAGT1 expression in breast cancer cells with qPCR and western blot.

**Figure:**
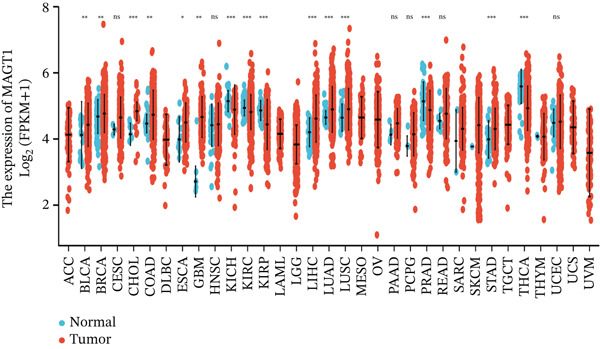
(a)

**Figure:**
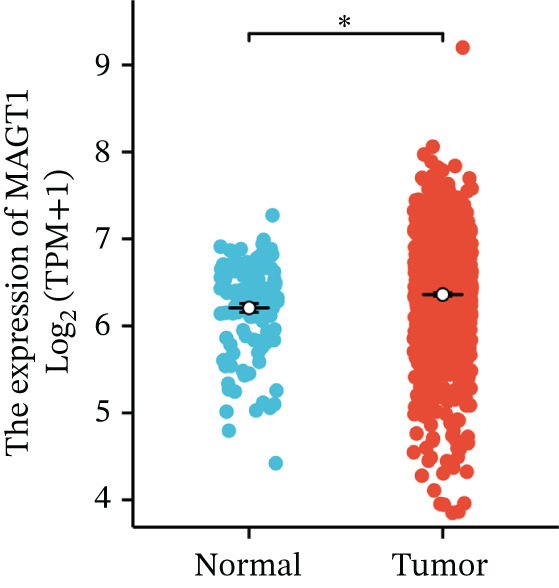
(b)

**Figure:**
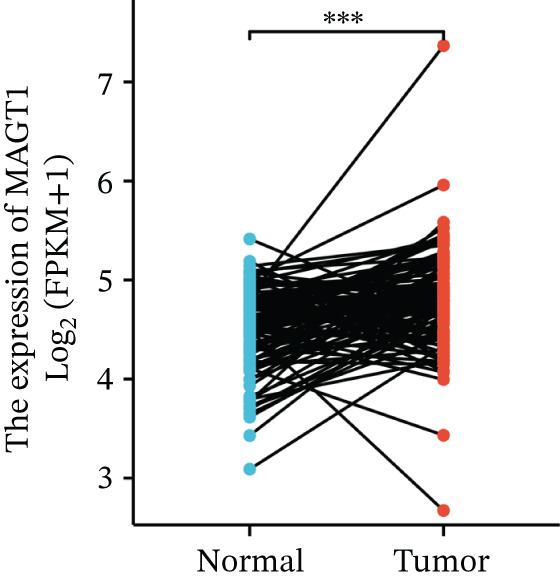
(c)

**Figure:**
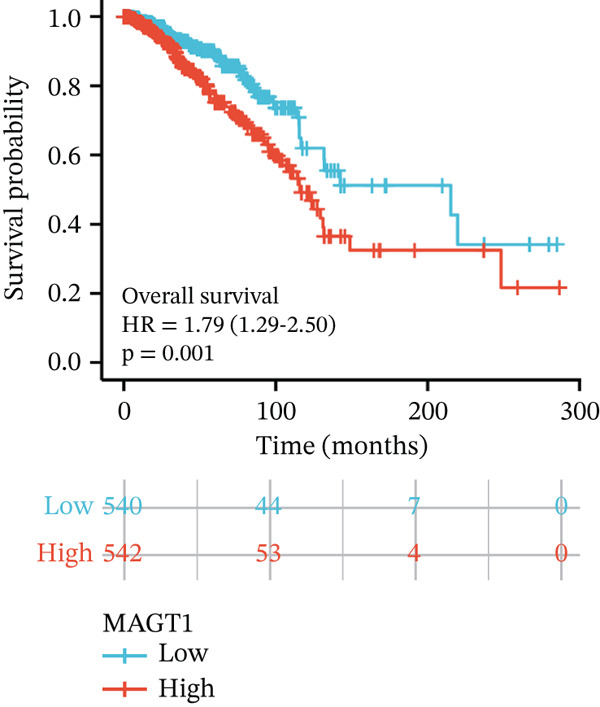
(d)

**Figure:**
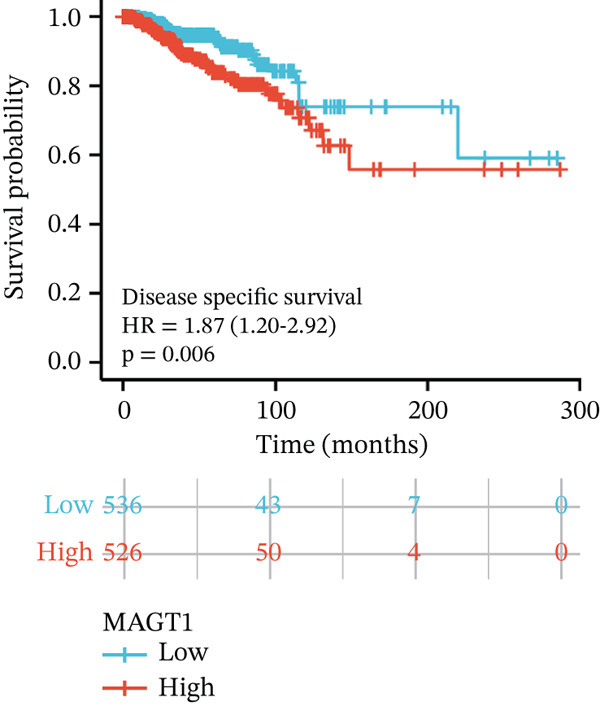
(e)

**Figure:**
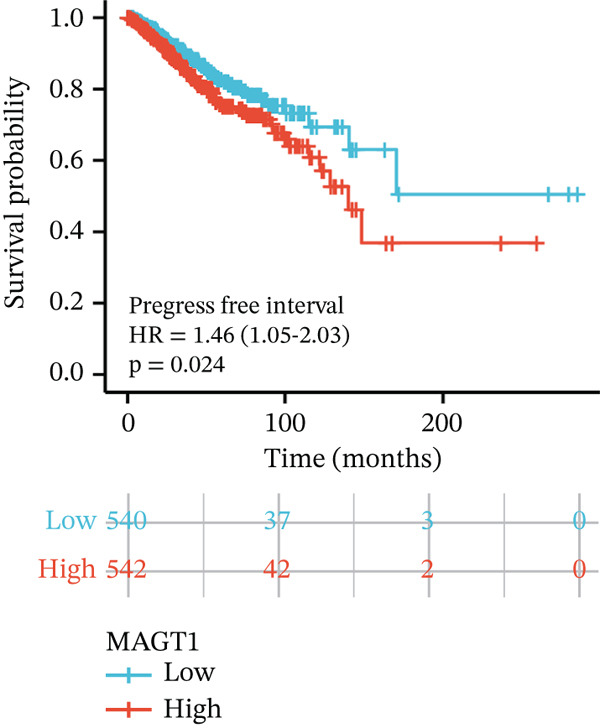
(f)

**Figure:**
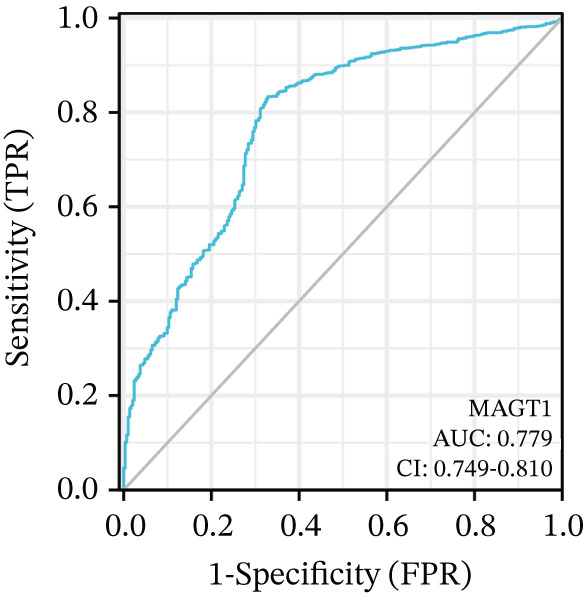
(g)

**Figure:**
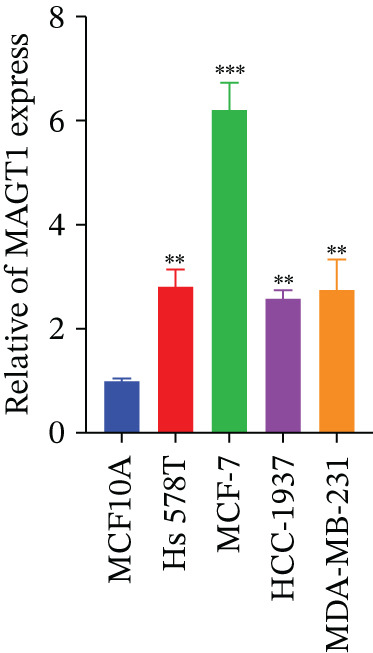
(h)

**Figure:**
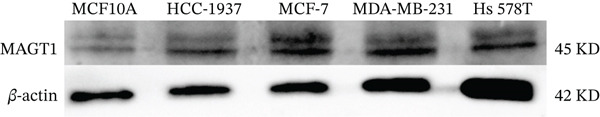
(i)

### 3.2. Relationship Between MAGT1 and BRCA Molecular Subtypes

We found that MAGT1 was associated with estrogen receptor expression and BRCA molecular subtypes. Further analysis revealed that low MAGT1 expression affected the OS of patients with estrogen receptor, progesterone receptor, and HER2 status (Figure 2a, 2b, 2c), as well as Luminal A patients (Figure 2d), but not statistically significant for Luminal B, HER2+, and TNBC (Figure 2e, 2f, 2g). Additionally, the correlation between MAGT1 expression and major clinical pathological factors, including age (≤ 60 vs. > 60, *p* = 0.559), T classification (T3 and T4 vs. T1 and T2, *p* = 0.032), lymph node status (N1 and N2 and N3 vs. N0, *p* = 0.852), distant metastasis (M1 vs. M0, *p* = 0.24), pathological stage (Stage III and Stage IV vs. Stage I and Stage II, *p* = 0.568), ER (Positive vs. Negative, *p* = 0.045), PR (Positive vs. Negative, *p* = 0.158), HER2 (Positive vs. Negative, *p* = 0.101), and molecular subtype PAM50 (Her2 and Basal vs. LumA and LumB, *p* = 0.048) were further determined using logistic regression, as shown in Table 1, indicating that MAGT1 expression was associated with poorer clinical pathological factors.

Figure 2Association between the MAGT1 expression levels and subtype with breast cancer. (a) ER status, (b) PR status, (c) HER2 status, (d) Lumainal A subtype, (e) Lumainal B subtype, (f) HER2 subtype, and (g) TNBC subtype.

**Figure:**
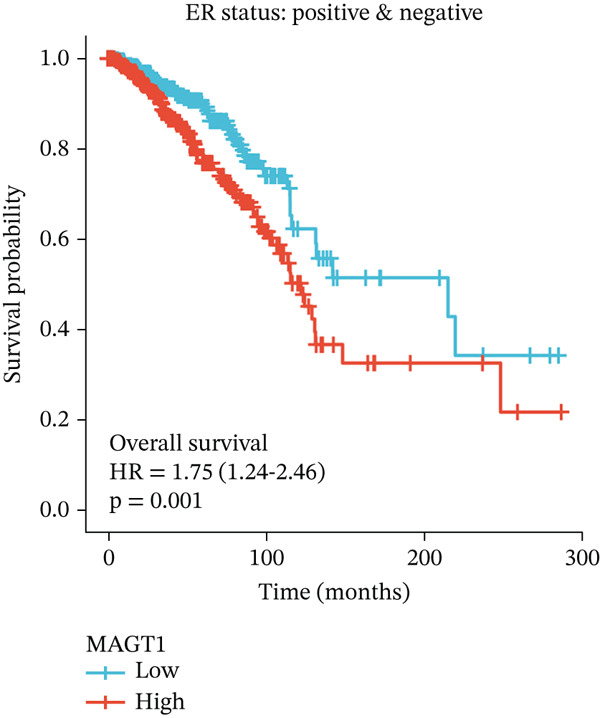
(a)

**Figure:**
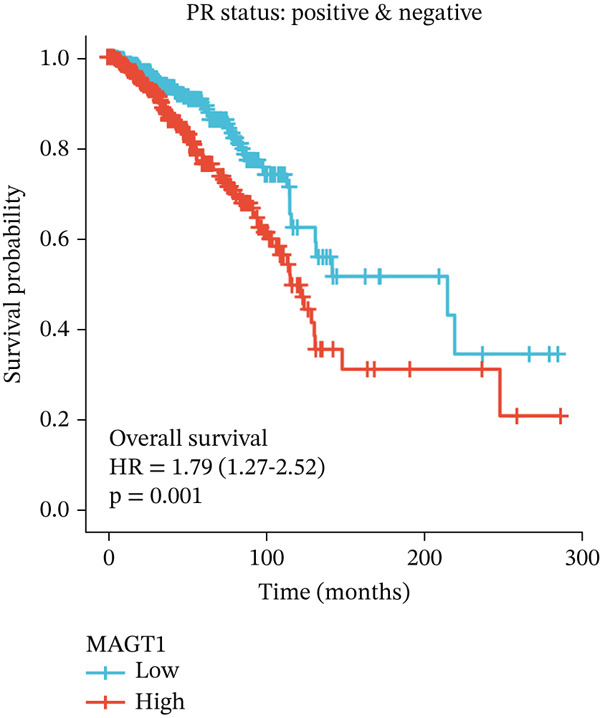
(b)

**Figure:**
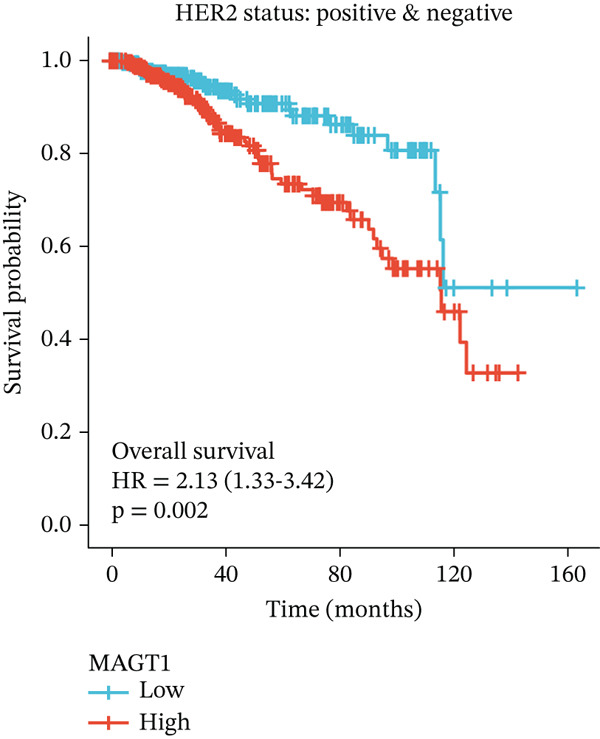
(c)

**Figure:**
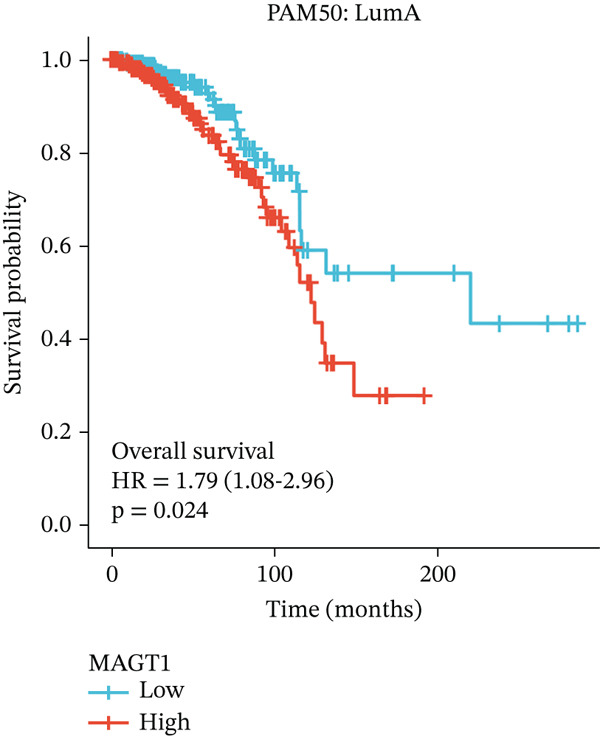
(d)

**Figure:**
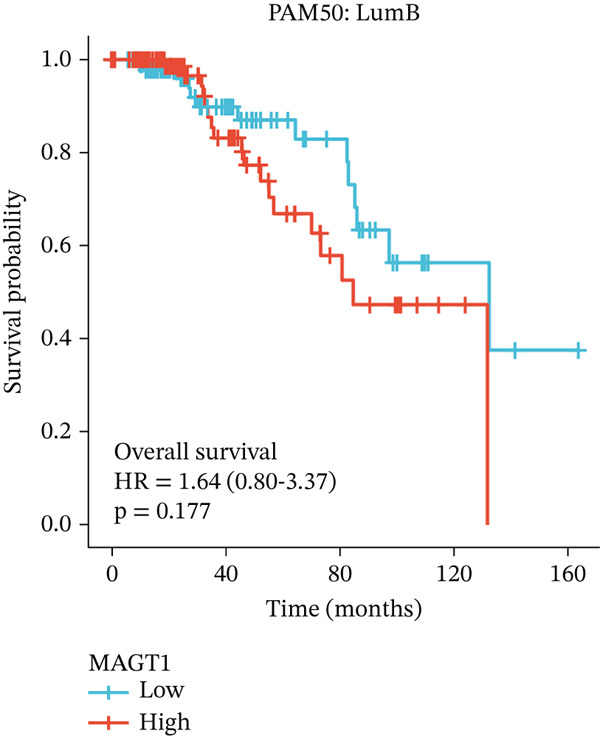
(e)

**Figure:**
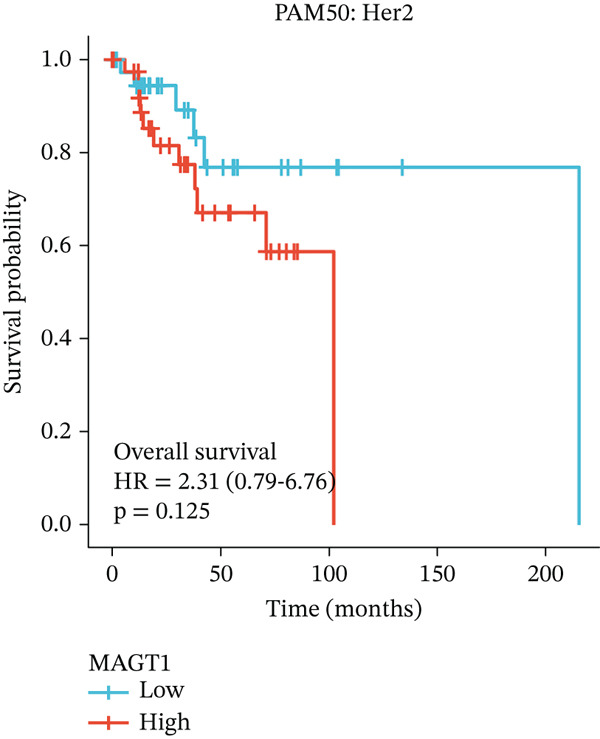
(f)

**Figure:**
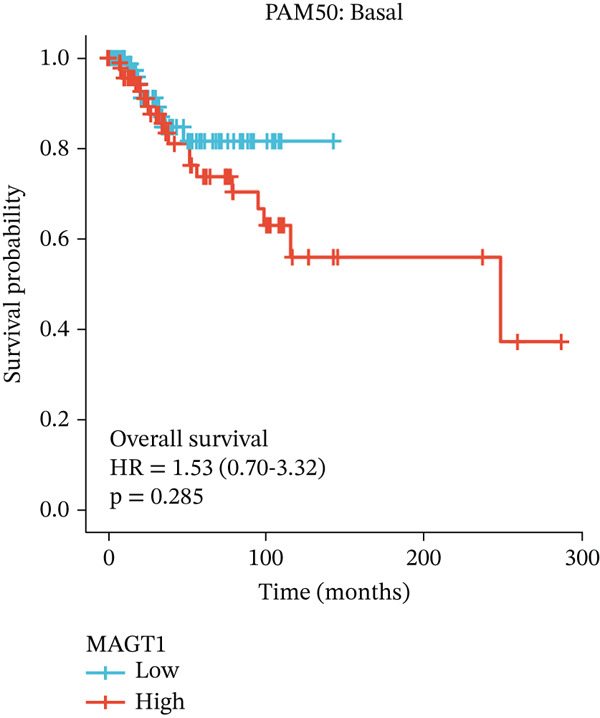
(g)

**Table 1:** Association of MAGT1 expression with clinical pathological characteristics by logistic regression.

Characteristics	Total (N)	Odds Ratio (OR)	*p* value
Age (> 60 vs. ≤ 60)	1.083	1.074 (0.845–1.365)	0.559
T stage (T3 and T4 vs. T1 and T2)	1.080	0.699 (0.502–0.968)	0.032
N stage (N1 and N2 and N3 vs. N0)	1.064	0.977 (0.768–1.243)	0.852
M stage (M1 vs. M0)	922	1.745 (0.708–4.682)	0.240
Pathologic stage (Stage III and Stage IV vs. Stage I and Stage II)	1.060	0.922 (0.696–1.219)	0.568
ER status (positive vs. negative)	1.033	0.743 (0.55–0.992)	0.045
PR status (positive vs. negative)	1.030	0.829 (0.639–1.075)	0.158
HER2 status (positive vs. negative)	715	1.349 (0.945–1.935)	0.101
PAM50 (Her2 and Basal vs. LumA and LumB)	1.043	1.321 (1.003–1.742)	0.048

Next, Cox regression models were utilized to simultaneously calculate the univariate and multivariate hazard ratios (HRs) for BRCA. As shown in Table 2, the univariate Cox regression analysis revealed that MAGT1 expression was a predictor of OS prognosis in BRCA patients (*H* *R* = 1.608, 95% CI 1.178–2.193, *p* = 0.003). Furthermore, the multivariate Cox regression analysis demonstrated that after adjusting for age, T classification, pathological grade, lymph node metastasis, distant metastasis, and molecular subtype, MAGT1 expression level remained an independent determinant factor influencing the OS prognosis of BRCA patients (HR = 1.739, 95% CI 1.099–2.751, *p* = 0.018).

**Table 2:** Associations between overall survival and clinicopathological characteristics in patients in TCGA using Cox regression.

Characteristics	Total (N)	Univariate analysis		Multivariate analysis	
		Hazard ratio (95% CI)	*p* value	Hazard ratio (95% CI)	*p* value
Age	1082				
≤ 60	601	Reference			
> 60	481	2.020 (1.465–2.784)	< 0.001	3.302 (1.979–5.507)	< 0.001
Pathologic stage	1059				
Stage I	180	Reference			
Stage II	619	1.699 (0.986–2.926)	0.056	1.106 (0.346–3.534)	0.865
Stage III and Stage IV	260	3.596 (2.059–6.280)	< 0.001	2.175 (0.397–11.908)	0.370
T stage	1079				
T1	276	Reference			
T2	629	1.332 (0.887–1.999)	0.166	0.975 (0.422–2.256)	0.954
T3 and T4	174	1.953 (1.221–3.123)	0.005	2.140 (0.773–5.930)	0.143
N stage	1063				
N0	514	Reference			
N1	357	1.956 (1.329–2.879)	< 0.001	1.401 (0.669–2.933)	0.371
N2 and N3	192	3.031 (1.939–4.739)	< 0.001	1.451 (0.437–4.816)	0.543
M stage	922				
M0	902	Reference			
M1	20	4.254 (2.468–7.334)	< 0.001	3.316 (1.187–9.264)	0.022
HER2 status	715				
Negative	558	Reference			
Positive	157	1.593 (0.973–2.609)	0.064	0.930 (0.480–1.802)	0.830
PAM50	1042				
LumA	561	Reference			
LumB	204	1.663 (1.088–2.541)	0.019	1.404 (0.737–2.676)	0.302
Basal	195	1.285 (0.833–1.981)	0.257	1.864 (0.980–3.545)	0.058
Her2	82	2.261 (1.325–3.859)	0.003	2.040 (0.876–4.749)	0.098
MAGT1	1082	1.608 (1.178–2.193)	0.003	1.739 (1.099–2.751)	0.018

### 3.3. MAGT1 Gene Mutation and Clinical Implications

We analyzed 32 TCGA cohorts from the cBioPortal database revealed MAGT1 alterations in approximately 1% of tumors, including missense mutations, splice site variants, truncating mutations, amplifications, structural rearrangements, and deep deletions (Figure S2A). In the TCGA pan‐cancer dataset, CNAs of MAGT1 were predominantly copy number losses, particularly in kidney chromophobe renal cell carcinoma and pheochromocytoma and paraganglioma (Figure S2C). The correlation between CNAs and MAGT1 mRNA expression in the BRCA dataset was less than 0.2 (Figure S2D). MAGT1 mutations were not statistically significantly associated with OS in BRCA (Figure S2E). In the TCGA BRCA cohort, patients were stratified into MAGT1‐high and MAGT1‐low expression groups based on the median mRNA expression level. The high‐expression group exhibited higher mutation frequencies in DMD, ERBB2, CREBBP, XIRP2, and RB1, whereas the low‐expression group showed higher mutation frequencies in CDH1, MYH9, and CUBN (Figure S2F). A comparative analysis of mutation spectra between MAGT1‐high and MAGT1‐low BRCA tumors identified TP53, PIK3CA, and TTN as the most frequently comutated genes (Figure S2G,H).

### 3.4. MAGT1 as an Independent Risk Factor Influencing BRCA OS

Through univariate and multivariate Cox regression analysis on the TCGA dataset, MAGT1 emerged as an independent risk factor, influencing BRCA OS (Figure 3a,b). Based on the results of the multivariate Cox regression, we constructed a nomogram to better predict the 1‐, 3‐, and 5‐year survival probabilities of BRCA with MAGT1 and age (Figure 3c). Simultaneously, calibration curves for 1‐, 3‐, and 5‐year survival rates were plotted, demonstrating that the predicted lines were close to the ideal line (Figure 3d), validating the prognostic efficiency and clinical applicability of the nomogram.

Figure 3Construction of nomogram and clinical data validation. (a–b) Univariate analysis and multifactorial analysis of the MAGT1 and clinicopathologic features predicted OS in breast cancer patients. (c) Construction of nomogram. (d) Calibration plots of survival probabilities at 1, 3, and 5 years. (e–f) The volcano plot and heat map show the relevant differentially expressed genes of MAGT1 in breast cancer tissues.

**Figure:**
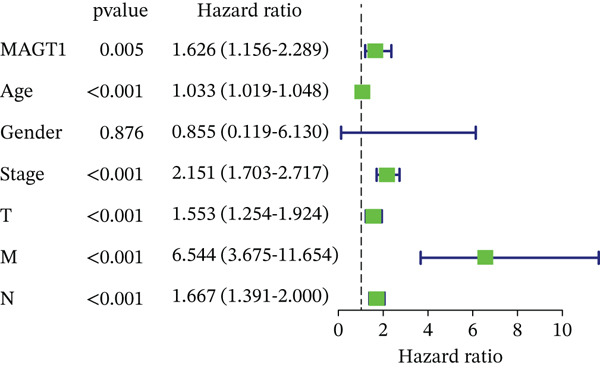
(a)

**Figure:**
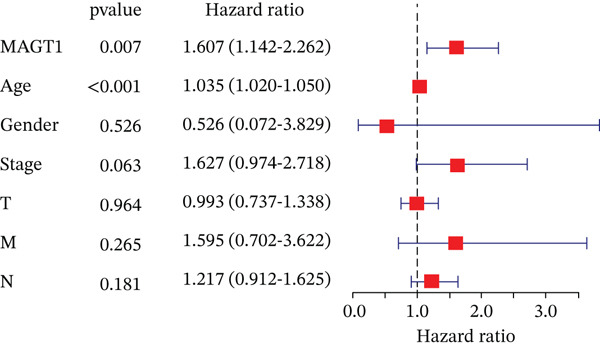
(b)

**Figure:**
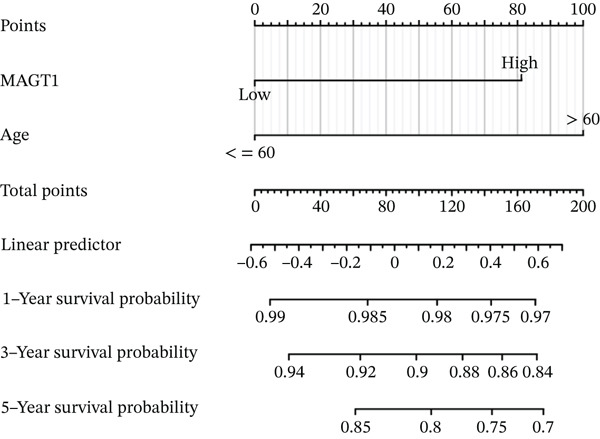
(c)

**Figure:**
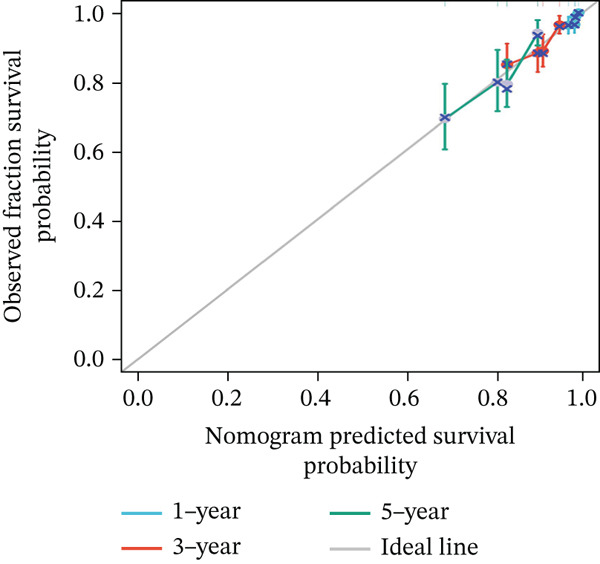
(d)

**Figure:**
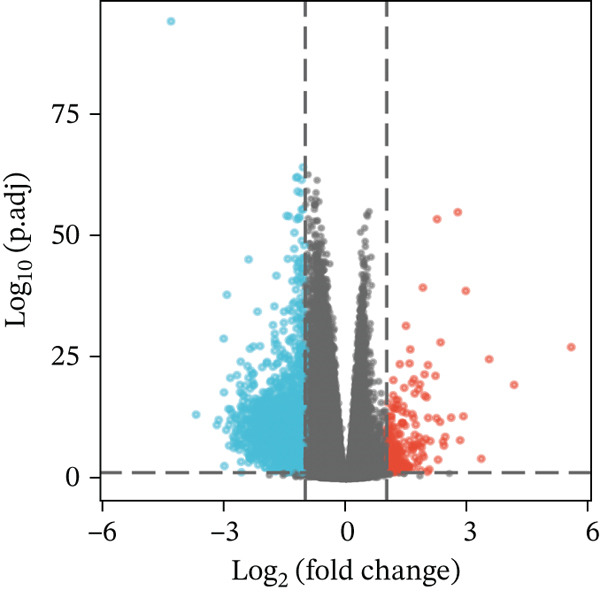
(e)

**Figure:**
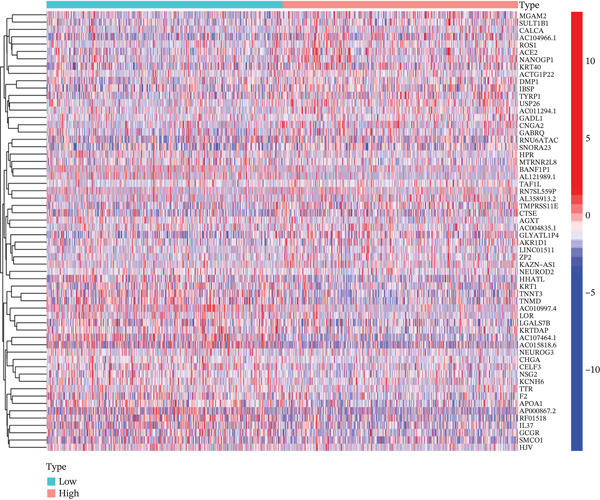
(f)

### 3.5. MAGT1‐Associated DEGs and Enrichment Analysis

Volcano plot (Figure 3e) and heat map (Figure 3f) were generated to visualize the differential expression of genes associated with MAGT1 in BRCA. Gene ontology (GO) functional enrichment, KEGG pathway enrichment, and GSEA were performed on the differentially expressed genes. In biological processes (BP), negative regulation of amine transport and glucose metabolism were predominantly enriched (Figure 4a), whereas in cellular components (CC), the conversion of high‐density/low‐density lipoprotein particles might be affected (Figure 4b). In molecular function (MF), association with hormone activity was significant (Figure 4c). KEGG pathway enrichment revealed the significant involvement of the pentose and glucuronate interconversions pathway (Figure 4d). GSEA enrichment indicated associations with drug metabolism, retinol metabolism, oxidative response, and cytochrome P450‐related drug metabolism (Figure 4e).

Figure 4The relevant differentially expressed genes of MAGT1 enrichment analysis. (a) GO enrichment with biological processes. (b) GO enrichment with cellular components. (c) GO enrichment with molecular function. (d) KEGG enrichment. (e) GSEA enriched.

**Figure:**
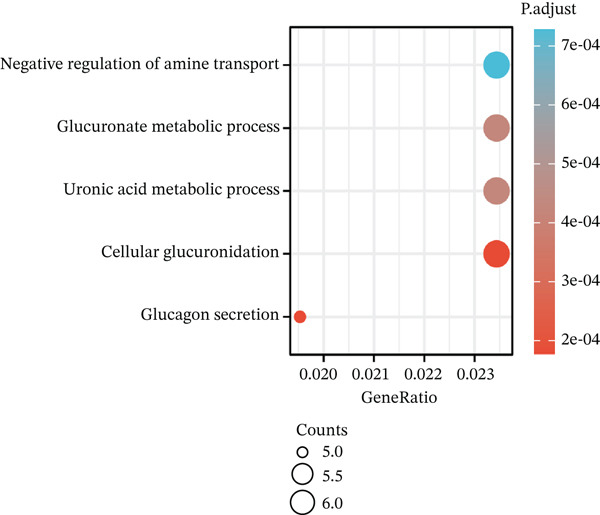
(a)

**Figure:**
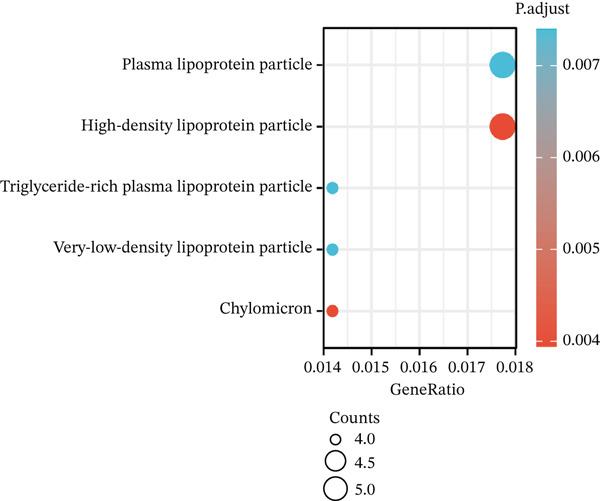
(b)

**Figure:**
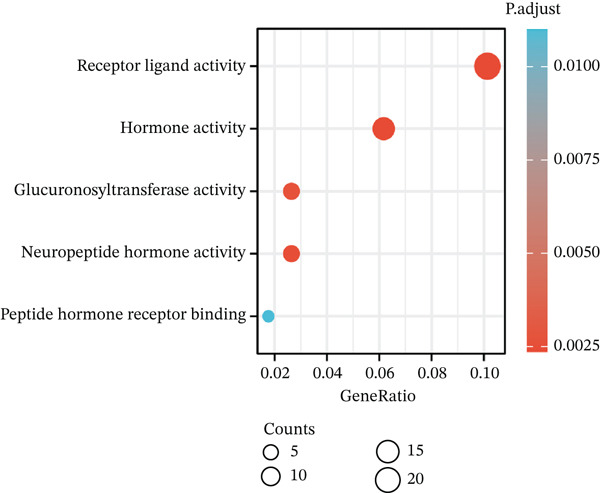
(c)

**Figure:**
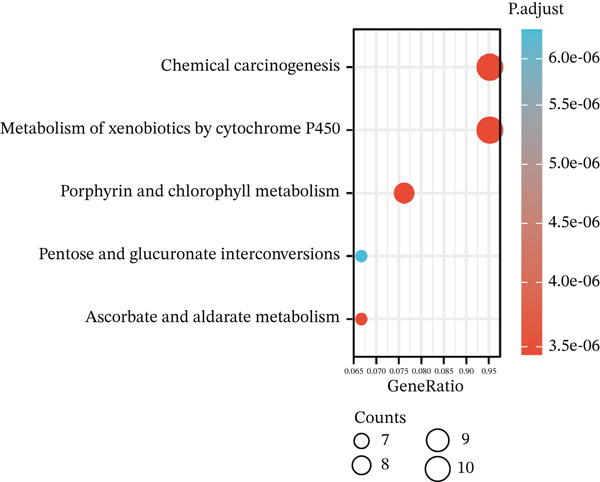
(d)

**Figure:**
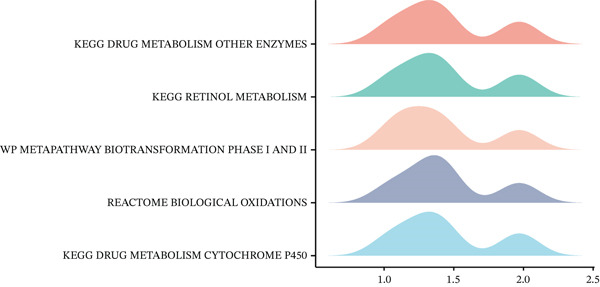
(e)

### 3.6. MAGT1 Correlation With Immune Infiltration in BRCA

Our results revealed a close association between MAGT1 expression and immune infiltration cells. In the low MAGT1 expression group, there was an increase in the proportion of CD8+ T cells, regulatory T cells (Tregs), activated NK cells, and resting mast cells. Conversely, in the high MAGT1 expression group, there was an increase in the proportion of activated CD4+ memory T cells and M1 macrophages (Figure 5a). MAGT1 showed positive correlations with T helper cells (Th), central memory T cells (Tcm), and Th2 cells, whereas it exhibited negative correlations with plasmacytoid dendritic cells (pDCs), CD56dim NK cells, and NK cells (Figure 5b). Specifically, MAGT1 was positively correlated with Tcm cells (*R* = 0.434, *p* < 0.001), Th cells (*R* = 0.348, *p* < 0.001), and Th2 cells (*R* = 0.244, *p* < 0.001), whereas it was negatively correlated with pDCs (*R* = −0.378, *p* < 0.001), CD56bright NK cells (*R* = −0.202, *p* < 0.001), and NK cells (*R* = −0.11, *p* < 0.001) (Figure 5c, 5d, 5e, 5f, 5g, 5h).

Figure 5Association between MAGT1 and immune infiltration expression in BRCA. (a) Comparison of the immune cell fraction difference between the low and high MAGT1 expression groups. (b) Relative proportions of 22 subtypes of tumor‐infiltrating immune cells for each sample in BRCA by CIBERSORT. (c–h) Correlation of MAGT1 expression with infiltration levels of Tcm cells, Th cells, Th2 cells, pDCs, NK CD56 cells, and NK cells.

**Figure:**
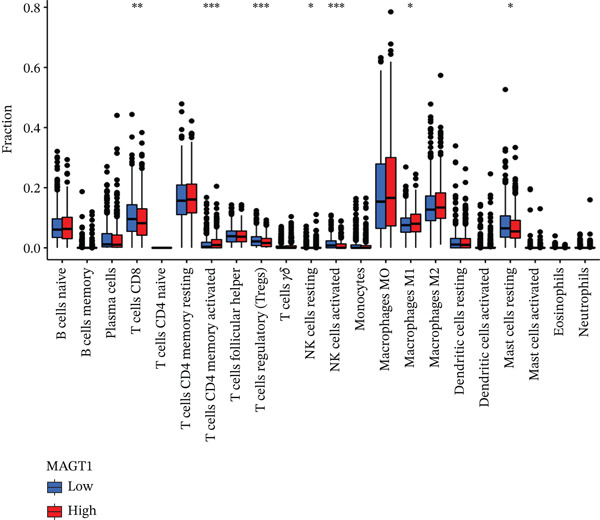
(a)

**Figure:**
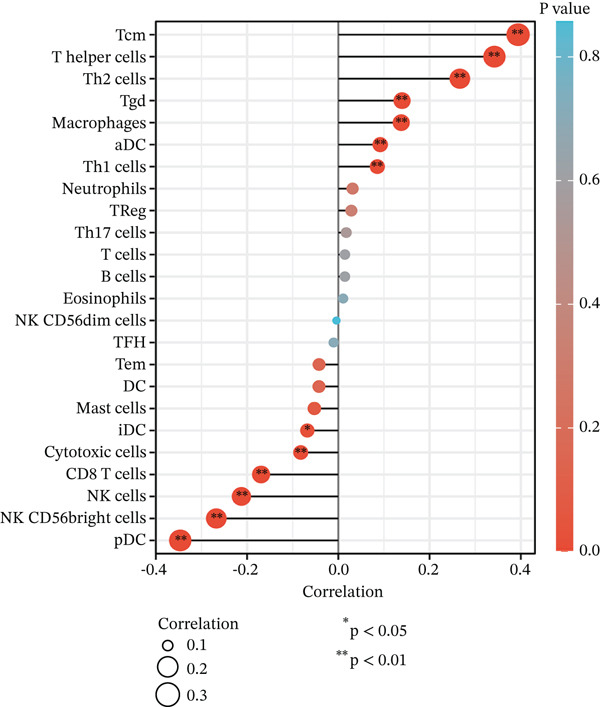
(b)

**Figure:**
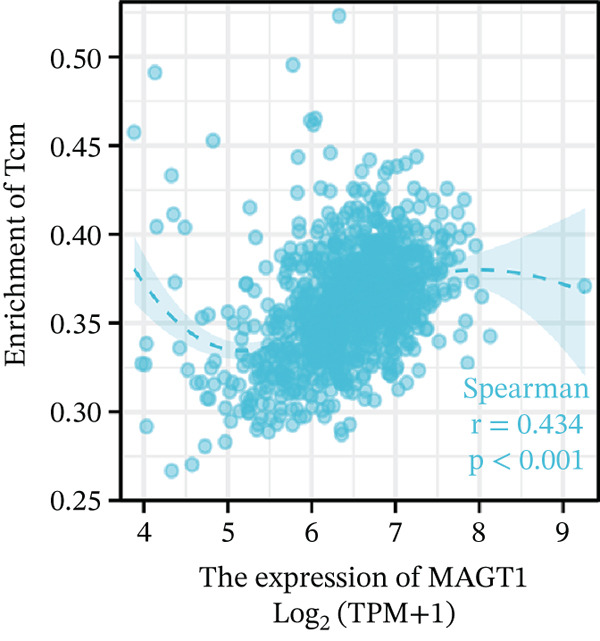
(c)

**Figure:**
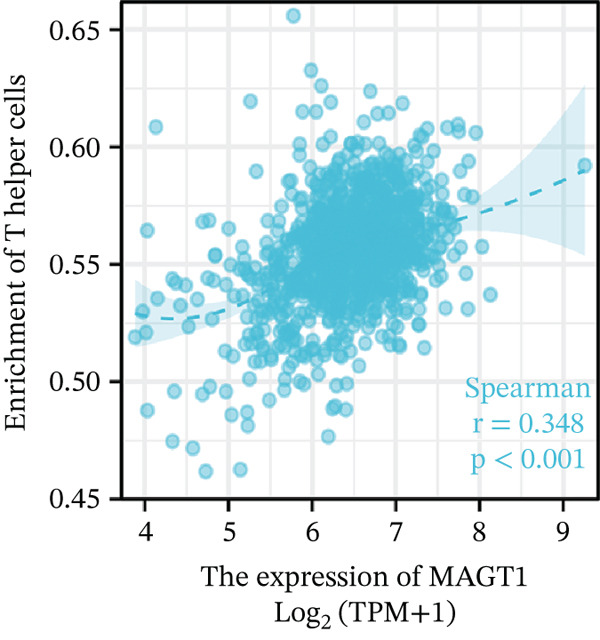
(d)

**Figure:**
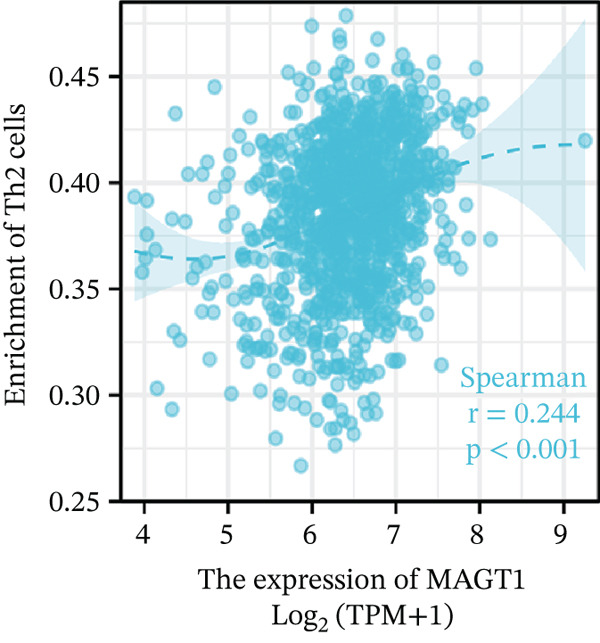
(e)

**Figure:**
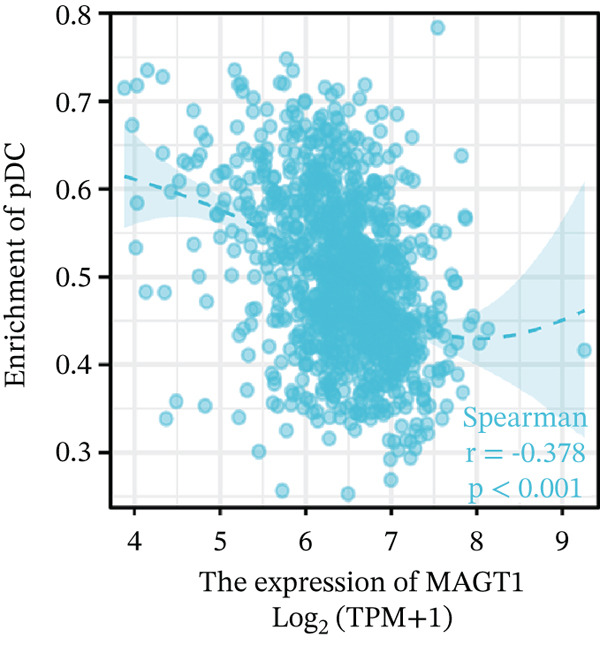
(f)

**Figure:**
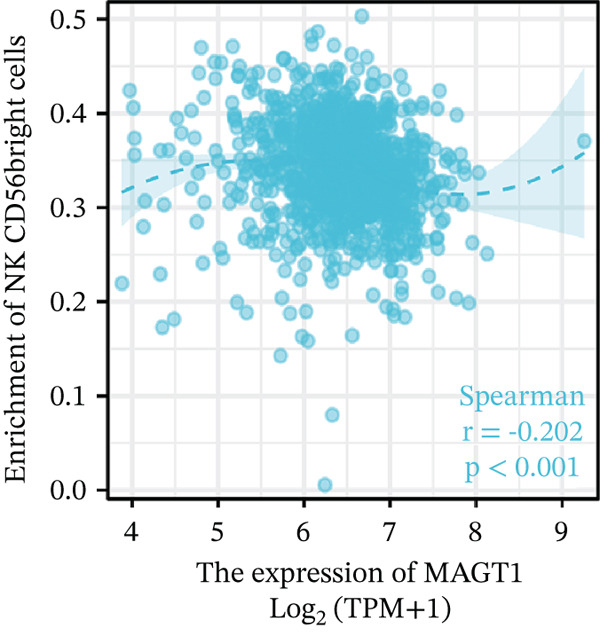
(g)

**Figure:**
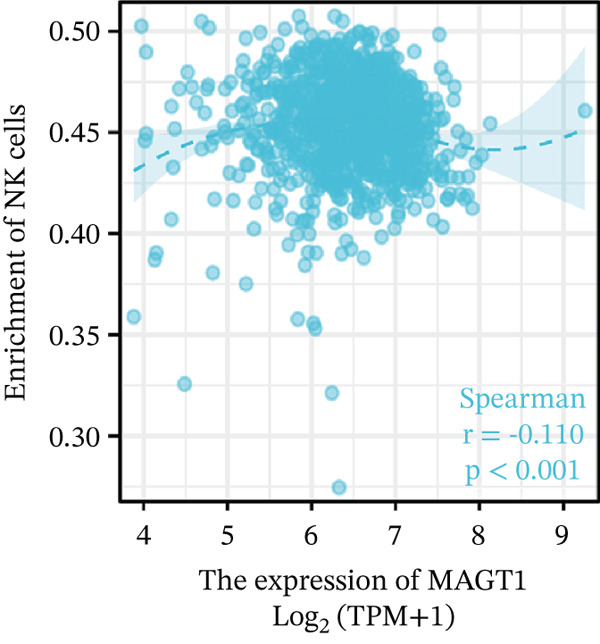
(h)

### 3.7. MAGT1 Influence on BRCA Cell Functions

The successful knockdown of MAGT1 in BRCA cells was confirmed by RT‐qPCR and western blot (Figure 6a,b). Subsequent experiments were conducted using siRNA‐2 and siRNA‐3. MTT assay results demonstrated that knocking down of MAGT1 significantly inhibited the proliferative viability of MCF‐7 and MDA‐MB‐231 cells (Figure 6c). Scratch assay results showed that downregulation of MAGT1 could significantly inhibit the migration capacity of MCF‐7 and MDA‐MB‐231 cells (Figure 6d). Plate cloning assay confirmed that downregulation of MAGT1 could inhibit the colony formation of BRCA cells (Figure 6e). Additionally, the EdU experiment showed that downregulation of MAGT1 could inhibit the DNA replication ability of cells (Figure 6f). Transwell experiments confirmed that downregulation of MAGT1 inhibited the migration and invasion capacity of MCF‐7 and MDA‐MB‐231 cells (Figure 6g).

Figure 6MAGT1 is involved in cell proliferation and migration in breast cancer cells. (a–b) The qPCR and western blot confirmed the expression of MAGT1with siRNA. (c) Effect of downexpression MAGT1 on the proliferative activity of MCF‐7 and MDA‐MB‐231 cells. (d) Downexpression MAGT1 effect on the migration ability of MCF‐7 and MDA‐MB‐231 cells. (e) Downexpression MAGT1 effect on the clonal formation of MCF‐7 and MDA‐MB‐231 cells. (f) MAGT1 affect the DNA replication ability of MCF‐7 and MDA‐MB‐231 cells. (g) Downexpression MAGT1 effect the migration and invasion of MCF‐7 and MDA‐MB‐231 cells.

**Figure:**
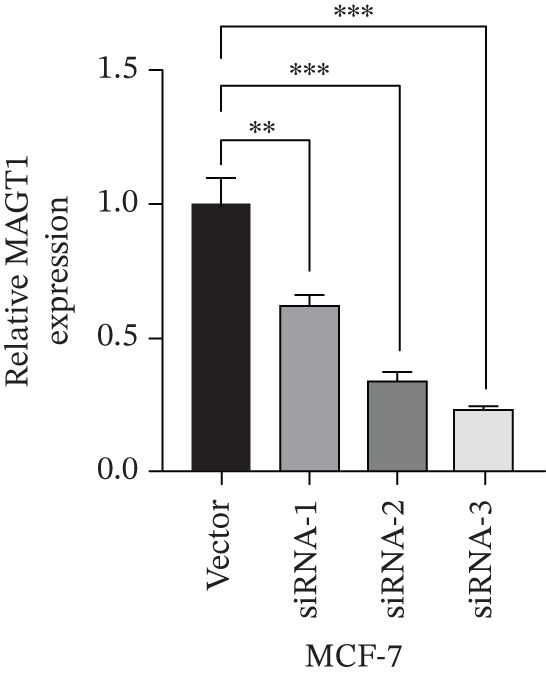
(a)

**Figure:**
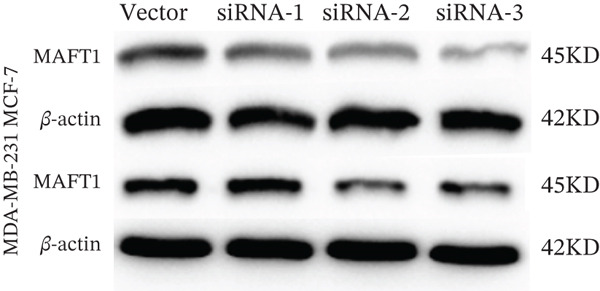
(b)

**Figure:**
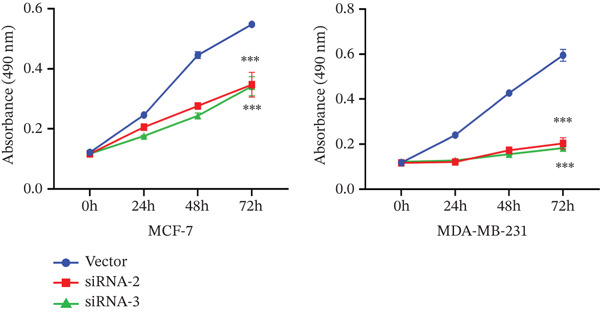
(c)

**Figure:**
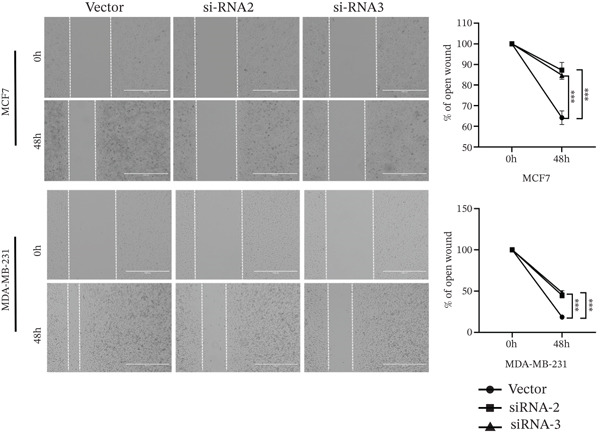
(d)

**Figure:**
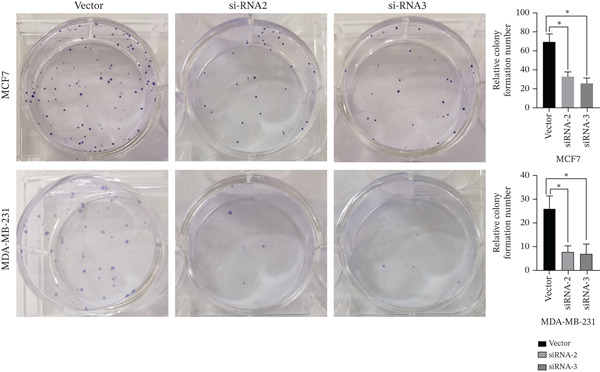
(e)

**Figure:**
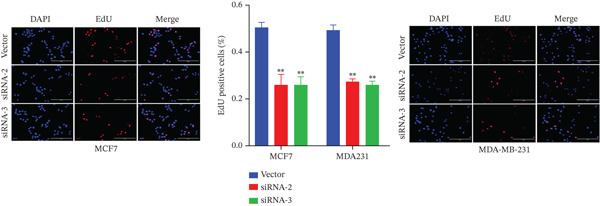
(f)

**Figure:**
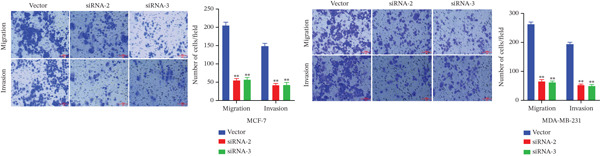
(g)

### 3.8. MAGT1 Expression in BRCA Tissues

**Figure 7:**
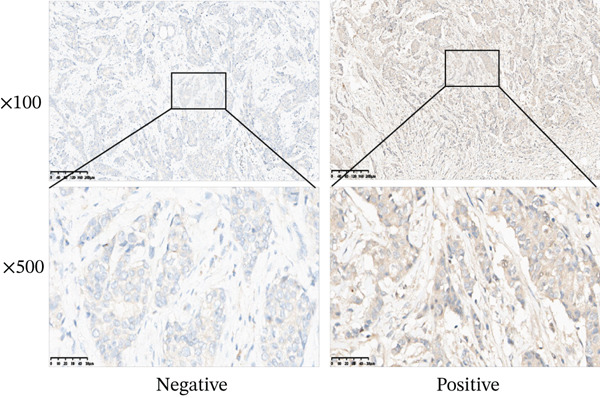
MAGT1 expression in breast cancer tissues. Immunohistochemical analysis of MAGT1 expression in breast tumor tissues (100× and 400× magnifcation).

Immunohistochemical analysis revealed that MAGT1 was expressed in both BRCA and adjacent tissues, with significantly higher expression in BRCA tissues (Figure 7). Analysis indicated correlations between MAGT1 expression and tumor T stage and histological grade in BRCA tissues (Table 3).

**Table 3:** The relationship between the positive and negative expression of MAGT1 and different clinical indicators in patients with breast cancer.

Characteristics	Positive	Negative	*p* value
*n*	49	11	
Age,	57.02 ± 10.894	53.182 ± 8.7272	0.280
≤ 60	34	9	
> 60	15	2	
T, *n* (%)			< 0.001
T1	9 (18.4%)	10 (90.9%)	
T2	36 (73.5%)	1 (9.1%)	
T3	4 (8.2%)	0 (0%)	
N, *n* (%)			0.158
N0	27 (55.1%)	10 (90.9%)	
N1	10 (20.4%)	1 (9.1%)	
N2	10 (20.4%)	0 (0%)	
N3	2 (4.1%)	0 (0%)	
M, *n* (%)			0.343
M0	41 (83.7%)	11 (100%)	
M1	8 (16.3%)	0 (0%)	
Stage, *n* (%)			< 0.001
Stage I	1 (2%)	10 (90.9%)	
Stage II	29 (59.2%)	1 (9.1%)	
Stage III	11 (22.4%)	0 (0%)	
Stage IV	8 (16.3%)	0 (0%)	
Grade, *n* (%)			< 0.001
I	0 (0%)	6 (54.5%)	
II	36 (73.5%)	3 (27.3%)	
III	13 (26.5%)	2 (18.2%)	
Molecular subtypes			
Luminal A	21 (42.9%)	5 (45.5%)	0.093
Luminal B	13 (26.5%)	3 (27.3%)	
HER2	6 (12.2%)	1 (9%)	
TNBC	9 (18.4%)	2 (18.2%)	

## 4. Discussion

BRCA incidence has been steadily increasing over the years, with contributing factors including improvements in screening, delayed age of first childbirth, hormonal imbalances, and obesity [17]. According to statistics, over 300,000 cases of BRCA are diagnosed in the United States annually, with nearly 80% being estrogen receptor‐positive (ER+) cases [18]. The majority of these patients rely on ER activation by the steroid hormone estrogen, and estrogen inhibition and antagonists have been the mainstay drugs for ER+ BRCA treatment [19]. In our study, we found a close association between low MAGT1 expression and survival in estrogen receptor and HER2 receptor subtypes, with better prognosis observed in Luminal A subtype with low MAGT1 expression. This suggests that MAGT1 expression in conjunction with estrogen levels in BRCA can better predict the prognosis of BRCA patients. HER2 is a highly glycosylated transmembrane receptor tyrosine kinase, and its N‐glycosylation is essential for proper protein folding, cell membrane localization, and signal transduction function [20]. Studies have shown that the extracellular domain of HER2 contains multiple N‐glycosylation sites, and altered glycosylation can influence its ligand‐binding affinity and downstream signaling activation [21]. As a subunit of the STT3B‐dependent oligosaccharyltransferase complex, MAGT1 specifically catalyzes the N‐glycosylation of cysteine‐proximal motifs (NCT/S) [22, 23]. Notably, analysis of the HER2 protein sequence reveals the presence of glycosylation sites that match the substrate characteristics of MAGT1. Thus, a reasonable hypothesis emerges: MAGT1 may influence HER2 maturation, membrane localization, and signal transduction function by modulating its glycosylation efficiency. If validated, this mechanism would provide a molecular basis for the observed association between high MAGT1 expression and enrichment of ERBB2 mutations. The MAGT1‐low expression group exhibited enrichment for CDH1 mutations, which are characteristic genomic alterations in invasive lobular carcinoma (typically ER+/HER2−) [24]. This observation implies a potential link between diminished MAGT1 expression and hormone receptor‐positive tumors of lobular histology. E‐cadherin, encoded by CDH1, is a critical regulator of epithelial polarity and intercellular adhesion; its functional loss serves as a defining feature of invasive lobular carcinoma [25]. As a heavily glycosylated transmembrane protein, E‐cadherin harbors multiple N‐glycosylation sites within its extracellular domain, and its glycosylation status modulates protein stability, membrane localization, and adhesive capacity [26]. Given that MAGT1 participates in the N‐glycosylation of cysteine‐proxified motifs, it is plausible that MAGT1 contributes to the glycosylation modification of E‐cadherin, thereby influencing its functional integrity [27]. This provides a potential mechanistic explanation for the observed association between low MAGT1 expression and enrichment of CDH1 mutations: in the context of reduced MAGT1 levels, E‐cadherin function may be compromised through dual mechanisms—genetic mutation and inadequate glycosylation—synergistically promoting the initiation and progression of invasive lobular carcinoma.

Although it is established that the DNA‐binding activity of p53 is zinc‐dependent rather than magnesium‐dependent, magnesium functions as an essential cofactor for various kinases and may indirectly influence p53 phosphorylation status. Studies have demonstrated that alterations in intracellular magnesium concentration can modulate the activity of ATM/ATR kinases, thereby affecting the p53‐mediated DNA damage response [28]. Consequently, MAGT1 may indirectly impact p53 function through the regulation of magnesium homeostasis. Recent investigations have revealed that MAGT1 knockdown suppresses AKT phosphorylation levels [28]. Notably, upstream receptors of the PI3K/AKT signaling pathway—including EGFR, HER2, and IGF‐1R—are heavily glycosylated transmembrane proteins. MAGT1 may indirectly regulate AKT activation by influencing the glycosylation maturation of these receptors.

In recent years, the tumor microenvironment (TME), particularly tumor‐infiltrating immune cells and cytokines, has been considered a key factor influencing cancer progression [29]. High immune infiltration is associated with improved clinical prognosis and treatment response in BRCA. Studies have found significant correlations between 12 types of immune cells in primary BRCA and OS of BRCA patients [30]. Predicting the impact of chemotherapy on BRCA patients using an immune scoring model has shown that patients in the low‐risk group have a more pronounced survival advantage. An immune scoring model based on immune cell infiltration can effectively and efficiently predict the prognosis of BRCA patients and the efficacy of chemotherapy [30, 31]. Research has shown that MAGT1‐dependent glycosylation is sensitive to Mg^2+^ levels, and a decrease in Mg^2+^ levels can impair immune cell function by affecting specific glycoproteins, with defects in protein glycosylation and gene expression forming the basis of immune deficiencies caused by MAGT1 defects in hereditary diseases [32]. MAGT1 gene deficiency leads to reduced intracellular free magnesium concentrations, resulting in defective expression of the activating receptor NKG2D on NK cells and CD8+ T cells, thereby impairing their cytolytic activity against EBV‐infected target cells [33]. A study revealed that with prolonged duration of HBV infection, MAGT1 expression in patients′ CD8+ T cells progressively declines, leading to reduced intracellular free magnesium concentrations. Consequently, this downregulation results in increased expression of the inhibitory receptor PD‐1 and decreased expression of the activating receptor NKG2D, ultimately culminating in functional exhaustion of CD8+ T cells. Furthermore, clinical intervention studies have shown that magnesium supplementation can restore MAGT1 expression levels, upregulate NKG2D while downregulating PD‐1, and partially reverse T cell exhaustion. These findings suggest that in the tumor context, downregulated MAGT1 expression may similarly induce magnesium deficiency in locally infiltrating CD8+ T cells, promoting their functional exhaustion through dysregulated PD‐1/NKG2D expression balance, thereby facilitating tumor immune evasion [6]. Our study also found a negative correlation between MAGT1 and CD8 T cells and NK cells, indicating that MAGT1 is involved in regulating immune infiltrating cells, affecting the progression of BRCA.

In cervical cancer research, it has been found that knocking down MAGT1 can inhibit cell proliferation by inducing S‐phase arrest and apoptosis in HeLa cells. Downregulation of MAGT1 can suppress the activation of ERK and p38, leading to the inhibition of tumor cell proliferation. The susceptibility of XMEN syndrome to EBV infection and defects in N‐linked glycosylation are increased due to mutations in the MAGT1 gene. Loss of MAGT1 function results in glycosylation defects, leading to the elimination of critical immune proteins such as the NKG2D receptor on CD8 T and NK cells, which is crucial for identifying and killing virus‐infected and transformed cells. Correcting the immunotoxicity of XMEN patient autologous lymphocytes by MAGT1 mRNA electroporation can effectively activate NKG2D, providing a new direction for XMEN disease treatment [31]. The mechanistic basis through which MAGT1 influences BRCA cell behavior remains to be fully elucidated. MAGT1 possesses two documented biochemical activities: it functions as a subunit of the STT3B‐containing oligosaccharyltransferase complex facilitating N‐glycosylation of cysteine‐proximal sites, and it has been implicated in cellular magnesium homeostasis [23]. Several lines of evidence suggest that the glycosylation‐related function may be particularly relevant to cancer biology. First, MAGT1 localizes predominantly to the endoplasmic reticulum rather than the plasma membrane, consistent with its role in OST complexes. Second, the protein′s thioredoxin‐like domain and CVVC active site motif are required for its oxidoreductase activity in glycosylation [34]. Emerging evidence links altered protein glycosylation to multiple cancer hallmarks, including proliferation, invasion, and immune evasion [35]. However, direct experimental evidence distinguishing these mechanisms in BRCA cells is currently lacking. We propose that future studies employing separation‐of‐function mutants, magnesium supplementation experiments, and glycoproteomic profiling will be essential to determine whether MAGT1 promotes BRCA progression primarily through its role in protein N‐glycosylation, through effects on magnesium homeostasis, or through integration of both functions. Such mechanistic understanding could inform therapeutic strategies targeting MAGT1 or its downstream effectors in BRCA.

In summary, bioinformatics analysis showed that the expression of MAGT1 was upregulated in BRCA, and the high expression of MAGT1 was associated with poor prognosis of BRCA. We further confirmed that the expression of MAGT1 is closely related to BRCA proliferation and invasion. In the future, further in vivo studies are needed to elucidate MAGT1′s role in BRCA invasion and metastasis. MAGT1 may serve as a target gene for predicting BRCA prognosis.

## Author Contributions

Zhe Song designed the project. Li‐wen Zhao wrote the paper, performed bioinformatics analysis, and rigorously revised the final manuscript. Li‐wen Zhao and Zhe Song completed the MTT, colony forming, Transwell, western blot, qPCR and immunohistochemical.

## Funding

This study was supported by The Scientific Research Program of Hunan Provincial Health Commission (NO. D202304088350),

## Disclosure

All authors also read and agree to release versions of the manuscript.

## Ethics Statement

The institutional review board approved a waiver of informed consent for this study as it involved retrospective analysis of archived, anonymized tissue specimens, which presented no risk to the patients.

## Consent

The data obtained from the tissue microarrays of 60 breast cancer patients in the manuscript were submitted anonymously and did not require the signing of informed consent. All methods were carried out in accordance with relevant guidelines and regulations.

## Conflicts of Interest

The authors declare no conflicts of interest.

## Supporting information


**Supporting Information** Additional supporting information can be found online in the Supporting Information section. (Supporting Information) Figure S1: The MAGT1 expression is associated with poor prognosis GSE131769 (A), GSE47994 (B), and GSE42568 (C). Figure S2: Genomic landscape of MAGT1 in human cancers. (A) cBioPortal overview of MAGT1 alteration frequency and mutation categories across TCGA tumors. (B) Bar plot of MAGT1 alteration rates by cancer type. (C) In the TCGA pan‐cancer dataset, copy number variations of MAGT1 are predominantly copy number deletions. (D) Spearman correlation between copy‐number variation (CNV) and MAGT1 expression across cohorts in the GSCA database. (E) Kaplan–Meier curves for BRCA comparing overall survival (OS) in patients with versus without MAGT1 alterations. (F) In the TCGA breast cancer dataset, patients were divided into MAGT1‐high and MAGT1‐low expression groups based on the median mRNA expression level. (G–H) Waterfall plots depicting the 15 most frequently mutated genes in BRCA samples with high versus low MAGT1 expression.

## Data Availability

Data sharing is not applicable to this article as no datasets were generated or analyzed during the current study. The TCGA database (TCGA [https://portal.gdc.cancer.gov]), which includes RNA sequencing data from 1098 breast cancer samples and 113 healthy breast samples.
