# Supplementary material for: Integrative Genomic Analysis Identifies MAGT1 as a Key Regulator of Proliferation and Poor Prognosis in Breast Cancer
Source: Hum Mutat. 2026 Apr 17;2026:8673018. doi: 10.1155/humu/8673018 (PMC13087893; doi:10.1155/humu/8673018)
Supplement: Supplementary file 1 — Supporting Information Additional supporting information can be found online in the Supporting Information section. (Supporting Information) Figure S1: The MAGT1 expression is associated with poor prognosis GSE131769 (A), GSE47994 (B), and GSE42568 (C). Figure S2: Genomic landscape of MAGT1 in human cancers. (A) cBioPortal overview of MAGT1 alteration frequency and mutation categories across TCGA tumors. (B) Bar plot of MAGT1 alteration rates by cancer type. (C) In the TCGA pan‐cancer dataset, copy number variations of MAGT1 are predominantly copy number deletions. (D) Spearman correlation between copy‐number variation (CNV) and MAGT1 expression across cohorts in the GSCA database. (E) Kaplan–Meier curves for BRCA comparing overall survival (OS) in patients with versus without MAGT1 alterations. (F) In the TCGA breast cancer dataset, patients were divided into MAGT1‐high and MAGT1‐low expression groups based on the median mRNA expression level. (G–H) Waterfall plots depicting the 15 most frequently mutated genes in BRCA samples with high versus low MAGT1 expression. [file HUMU-2026-8673018-s001.docx]

**Figure legends**

**
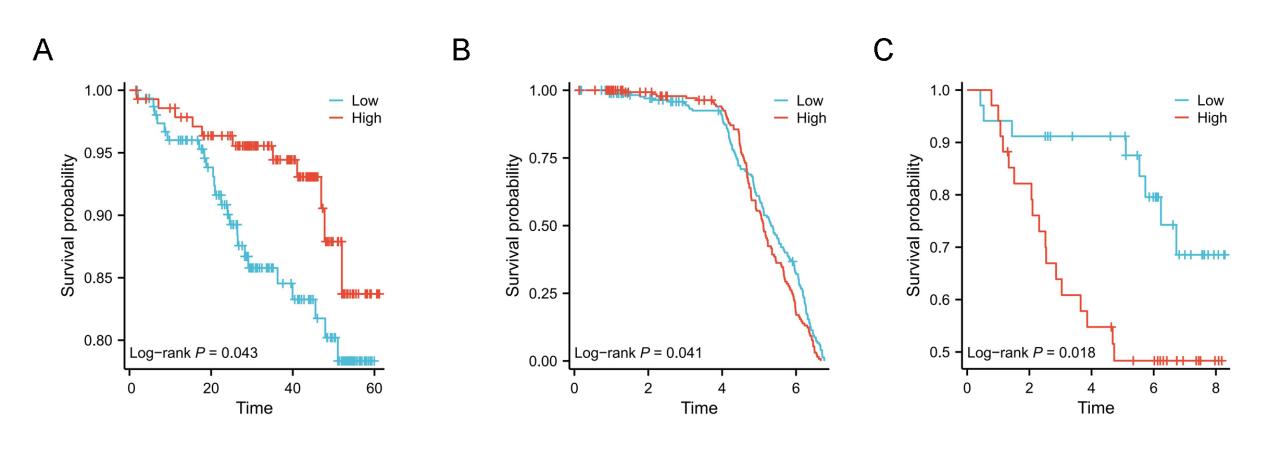
**

Supplementary Figure 1: The MAGT1 expression is associated with poor prognosis GSE131769(A), GSE47994 (B), and GSE42568(C).


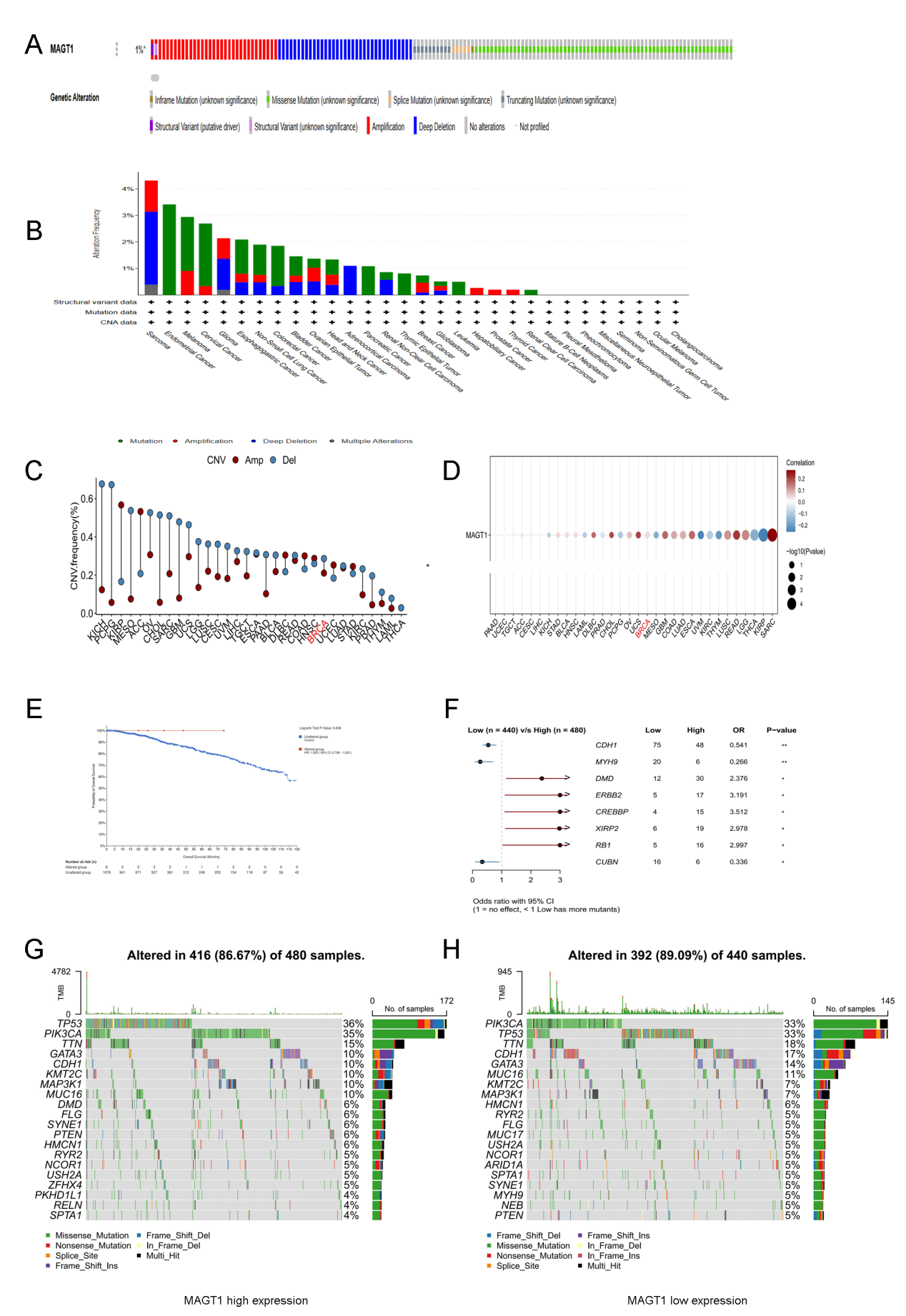


Supplementary Figure 2: Genomic landscape of MAGT1 in human cancers. (A) cBioPortal overview of MAGT1 alteration frequency and mutation categories across TCGA tumours. (B) Bar plot of MAGT1 alteration rates by cancer type. (C) In the TCGA pan-cancer dataset, copy number variations of MAGT1 are predominantly copy number deletions. (D) Spearman correlation between copy-number variation (CNV) and MAGT1 expression across cohorts in the GSCA database. (E) Kaplan–Meier curves for BRCA comparing overall survival (OS) in patients with versus without MAGT1 alterations. (F) In the TCGA breast cancer dataset, patients were divided into MAGT1-high and MAGT1-low expression groups based on the median mRNA expression level. (G-H)Waterfall plots depicting the 15 most frequently mutated genes in BRCA samples with high versus low MAGT1 expression.
